# *Acanthamoeba* Keratitis Secondary Glaucoma Associated With Mature Cataract and a Fixed Dilated Pupil in a 40-Eye Series

**DOI:** 10.1097/ICO.0000000000003918

**Published:** 2025-06-19

**Authors:** Rohan Hussain, John K. G. Dart, Sana Hamid

**Affiliations:** * Moorfields Eye Hospital NHS Foundation Trust, London, United Kingdom; and; † National Institute of Health Research (NIHR) Moorfields Biomedical Research Centre, UCL Institute of Ophthalmology, London, United Kingdom.

**Keywords:** *Acanthamoeba* keratitis, secondary glaucoma, risk factors, outcomes

## Abstract

**Purpose:**

This case series describes the incidence, clinical associations, and treatment outcomes of *Acanthamoeba* keratitis (AK) secondary glaucoma to identify potential prophylactic measures and optimal treatment.

**Methods:**

AK-affected eyes developing secondary glaucoma from 1992 to 2020 were identified from Moorfields databases. The annualized incidence was established from those patients with AK registered between 2000 and 2015.

**Results:**

The 2000–2015 incidence of AK secondary glaucoma was 26 of 417 (6.2%). Forty eyes (39 patients) developed glaucoma or ocular hypertension; 16 of 28 (57%) had been treated for AK for ≥12 months from onset. Thirty-four of 40 eyes (85%) had an associated keratoplasty; 26 of 40 (65%) had a fixed dilated pupil and/or mature cataract. Sixteen of 40 (40%) underwent antiglaucoma drug treatment alone. Twenty-four of 40 (60%) eyes had surgical treatments including cyclodiode laser (2 eyes) leading to phthisis or evisceration, trabeculectomy (2 eyes) failed, glaucoma drainage devices in 20 of 40 (50%) eyes resulted in glaucoma control in 18/20 (90%) but required additional surgery in 9 of 20 (45%) eyes.

**Conclusions:**

Mature cataract or a fixed dilated pupil has not been previously identified as a cause of secondary glaucoma in AK. The implications are that both result in angle closure and that early surgery for maturing cataract, despite its complexity, might prevent the development of angle closure. However, the potential for better medical treatment to reduce the time to cure to less than 12 months is likely the most effective way to reduce AK glaucoma incidence. Successful management of AK glaucoma once developed probably requires use of a glaucoma drainage device.

## Abstract 2

Supplemental Digital Content is Available in the Text.

Glaucoma secondary *to Acanthamoeba* keratitis (AK) has been recognized since the first description of AK.^1^ Studies on this topic are summarized in Supplemental Digital Content 1 (see Supplemental Appendix, http://links.lww.com/ICO/B839), of which only 2 have focused on the issue of glaucoma complicating AK.^2,3^ The earliest study excluded patients having had therapeutic keratoplasty (TK) and described glaucoma developing in 6 of 20 (30%), of whom 4 became blind and 2 had developed peripheral anterior synechiae resulting in angle closure thought to result from inflammation rather than pupil block.^3^ The most recent description of glaucoma associated with AK comes from a retrospective review of 52 consecutive patients with AK (all unilateral), in which 17 of 52 eyes (33%) developed glaucoma. Of these, 10 of 17 (59%) had undergone a keratoplasty, and steroid use was shown to be the only statistically significant risk factor of glaucoma.^2^ In the latter study, contrary to the findings of other studies reporting glaucoma in AK, glaucoma had no effect on visual outcomes despite 13 of 17 eyes requiring glaucoma surgery. These 2 studies are from small case series and may have overestimated the proportions of patients with AK developing glaucoma. In an early (1993) consecutive series of 77 eyes with AK (72 patients), glaucoma developed in 10 of 77 (13%) and accounted for 5 of 15 patients (33%) with poor outcomes.^4^ TK for AK is known to have a poor prognosis^5^ and has recently been shown to have worse outcomes for AK than for other causes of infectious keratitis.^6^ Three studies on the outcomes of TK for AK included 66 patients (unilateral), of whom 24 (36%) developed secondary glaucoma,^7–9^ which is one of the reasons for the poor outcomes of TK for this disease.

In addition to the associations of TK, topical steroid use, and the possibility that angle closure from peripheral anterior synechiae may cause glaucoma in AK,^3^ there have been no other associations that might alert clinicians to the development of glaucoma. Furthermore, discussion of the management of glaucoma in the setting of AK has been limited to 1 study of 6 eyes, which concluded that “the current case series shows that medical therapy can be a temporary solution, but that in advanced AK, glaucoma drainage devices (GDDs) are needed.”^3^

Our report describes AK-associated secondary glaucoma in 40 eyes of 39 patients, the largest series to date. Its aims were to evaluate the incidence of glaucoma in AK, its clinical associations, and treatment outcomes. We have provided an incidence for the development of secondary glaucoma in AK. We have also identified unusual clinical features that might herald the development of AK-associated glaucoma, potential prophylactic measures that might prevent its onset and have data that supports a management plan for the condition when it develops.

## MATERIALS AND METHODS

This study was approved as a Moorfields Eye Hospital Clinical Audit (Reference 652). Anonymized patients with glaucoma or ocular hypertension (OHT) and AK were identified from a search of the hospital electronic patient records (started in 2000 and searched until June 2020) using the keywords *Acanthamoeba*, glaucoma, and OHT, together with a search of an earlier database for the period 1992–2000. The incidence of glaucoma or OHT in AK was calculated using as the denominator the total number of AK-affected eyes and patients who attended our center from 2000 to 2015, from a previously published data set of AK cases.^10^

A glaucoma or OHT diagnosis was defined as an intraocular pressure (IOP) of more than 21 mm Hg in the affected eye. Patients undergoing treatment for either condition were included. The association of OHT with glaucomatous optic neuropathy is difficult to determine in these patients because of corneal opacity, anterior chamber inflammation, and cataract, resulting in a poor fundal view for optic nerve assessment by fundoscopy or by optical coherence tomography. The presence of media opacity mean that visual field testing is usually not reliable or possible, hence the inclusion of both glaucoma and OHT in the data set.

Statistical analysis using linear regression was limited by the lack of clinical data at the time of glaucoma diagnosis and was restricted to a comparison of the outcomes between surgical treatment groups using the 2-sided Fisher exact test.

## RESULTS

Forty eyes with glaucoma or OHT were identified in 39 patients who developed AK between 1992 and 2020. Table 1 presents the demographics at the time of the onset of both AK and glaucoma and includes risk factors of a poor treatment outcome at the time of AK diagnosis. Twenty-eight of 35 eyes (80%) were from contact lens (CL) users. Twelve of 29 eyes (41%) were diagnosed with AK more than a month from symptom onset. The time from disease onset to a glaucoma diagnosis was ≥12 months in 16 of 28 eyes (57%). Thirty-four of 40 eyes (85%) had undergone a keratoplasty; of those with a known indication, over half [15/27 (56%)] were therapeutic. Four of 32 eyes (12.5%) were being treated with oral immunosuppression for severe *Acanthamoeba* scleritis at the time of glaucoma diagnosis.

**TABLE 1.** Demographics for 39 Patients and 40 Eyes Including Risk Factors of a Poor Outcome of AK Treatment and Clinical Characteristics at the Time of AK Diagnosis and Glaucoma Diagnosis, Respectively

Demographics	n (%)
**Total number of patients at the time of AK diagnosis**	39 patients
Age (yr)	
Median [range]	44.5 (17–80)
Unknown	1
Gender	
Male/female	17 (44)/22 (56)
Ethnicity	
White	32 (82)
Asian	4 (10)
Other (no Black or African Caribbean)	3 (8)
Eye affected	
OD/OS	18 (46)/20 (51)
Bilateral	1 (3)
**Total number of eyes at the time of AK diagnosis**	40 eyes
Previous contact lens wear*	
Yes/no	28 (80)/7 (20)
Unknown	5
**Risk factors of a poor treatment outcome at the time of AK diagnosis**	40 eyes
Topical steroid before antiamoebic treatment	
Yes/no	7 (41)/10 (59)
Unknown	23
Late diagnosis ≥30 d	
Yes/no	12 (41)/17 (59)
Unknown	11
**Clinical characteristics at the time of glaucoma diagnosis**	40 eyes
Time to glaucoma diagnosis from symptom onset	
Mean [standard deviation], d	918 (1907)†
Median [range], d	403 (19–8984)†
Numbers diagnosed with glaucoma at different periods from symptom onset	
≤1 mo	2 (7)
≥1 mo and ≤12 mo	10 (36)
≥12 mo	16 (57)
Unknown	12
**Keratoplasty (any type)**	34 eyes (85)
Therapeutic	15/27 (56)
Optical	12/27 (44)
Unknown indication	7
**Treatment of keratitis for all eyes at the time of glaucoma diagnosis**	32 eyes
Unknown	8
Nil	4 (13)
Topical antiamoebic treatment (AAT) only	7 (22)
Topical AAT with steroids	3 (9)
Topical AAT with steroids and oral nonsteroidal anti-inflammatory drugs (NSAIDs)	3 (9)
Oral immunosuppression for scleritis	4 (13)
Topical steroids only	9 (28)
Topical AAT and oral NSAIDs	1 (3)
Topical AAT and oral itraconazole	1 (3)

Cells contain n (%) unless otherwise specified in the row. Percentages are of valid totals excluding missing values.

* Types of CL in the 28 CL users: soft daily 9, soft monthly 7, rigid gas-permeable 2, soft biweekly 1, scleral 1.

† These numbers are skewed by 1 patient having AK in 1992 and developing glaucoma in 2017.

Supplemental Digital Content 2 (see Supplemental Table 1, http://links.lww.com/ICO/B840) provides the number of patients and eyes affected by AK for each year in the period 2000–2015 and provides a comparison of these with the number of eyes affected by glaucoma/OHT. Twenty-six of 417 eyes (6.2%) in 398 patients (19 having bilateral AK) developed glaucoma in this 16-year period; the proportions of AK eyes developing glaucoma varied from year to year (range 0%–28%). Supplemental Digital Content 3 (see Supplemental Figure 1, http://links.lww.com/ICO/B841) provides the annual incidence of 847 AK cases at Moorfields from 1984 to 2019. In the period 1992–2019 covered by this study, there were 794 patients with AK, of whom 38 developed glaucoma, an incidence of 4.8% based on patients, as opposed to eyes, with AK.

Table 2A summarizes the outcomes regarding best corrected visual acuity (BCVA) ≥6/60 and other key outcomes following the different treatments for glaucoma, and Table 2b presents the clinical findings associated with the development of secondary glaucoma for the 40-eye data set. Supplemental Digital Content 4 (see Supplemental Table 2, http://links.lww.com/ICO/B842) presents the complete data set from which this summary has been extracted:

- Outcomes (Table 2A): For all different glaucoma treatments combined, the proportion of eyes with BCVA ≥6/60 was 8 of 39 (21%). Antiglaucoma medications (AGMs) alone were used in 16 eyes, of which only 1 of 15 (7%) had a final BCVA of ≥6/60; 2 were eviscerated and 1 enucleated. Cyclodiode laser alone was used in 2 eyes, 1 of which developed phthisis and the other was eviscerated. Trabeculectomy was used in 2 eyes, 1 of which also received cyclodiode laser; both had poor visual outcomes, including 1 with no light perception due to uncontrolled pressure. Twenty eyes were treated with a GDD either alone (5), with the ripcord out (6), or combined with cyclodiode (9); of these, 7 of 20 (35%) had a final BCVA of ≥6/60 for which the individual acuity outcomes are given.
- Associated clinical findings (Table 2B): Topical steroids were used in 38 of 40 eyes (95%), and keratoplasty was performed in 34 of 40 (85%). Some clinical findings, otherwise uncommon in AK, were frequent in this cohort, without much difference between those treated medically and surgically: angle closure in 15 of 22 assessable eyes (68%), mature cataract alone in 8 of 40 (20%), a fixed dilated pupil alone (also known as iris atrophy and paralytic mydriasis) in 5 of 40 (13%), and mature cataract combined with a fixed dilated pupil in 13 of 40 eyes (33%). Eyes affected by mature cataract and/or a fixed dilated pupil made up 26 of 40 glaucoma/OHT cases (65%).

**TABLE 2.** Summary of Treatment Outcomes (A) and Associated Clinical Findings (B) in 40 Eyes (39 Patients) With AK-Associated Glaucoma

A		
Glaucoma Treatments			Total Number of Eyes (N)	Final BCVA Better Than/Equal to 6/60, N (Percent)	Other Key Outcomes
All treatments combined	40	8 (21)	3 eviscerated, 1 enucleated, 1 phthisical
*Missing data*		*1*	
AGM alone ± keratoplasty	16	1 (7)	2 eviscerated, 1 enucleated
*Missing data*		*1*	
Cyclodiode laser alone	2	0	1 eviscerated, 1 phthisical
Trabeculectomy ± cyclodiode	2	0	1 uncontrolled IOP leading to no perception of light (NPL)
GDDs	20	7 (35)	GDD included: GDD alone (n = 5); GDD with ripcord out (n = 6); and GDD with cyclodiode (n = 9) Individual final visual acuities: 6/9, 6/12, 6/15, 6/18, 6/18, 6/24, and 6/36 for those with a final BCVA ≥6/60

B	
Associated Clinical Findings in 40 Eyes		N (Percent)
Topical steroid use	38 (95)
Keratoplasty	34 (85)
Angle closure in assessable eyes	15 (68)
*Missing data*	*18*
Mature cataract alone	8 (20)
Fixed dilated pupil (also termed iris atrophy and paralytic mydriasis) alone	5 (13)
Mature cataract with fixed dilated pupil	13 (33)
Mature cataract and/or fixed dilated pupil	26 (65)

Percentages are of valid totals excluding missing values.

The complete data set from which this summary is derived is in Supplemental Digital Content 4 (see Supplemental Table 2, http://links.lww.com/ICO/B842), which includes a detailed breakdown of glaucoma treatment groups (all combined, AGM alone, and different glaucoma surgeries and their combinations, both with and without a keratoplasty) for outcomes of visual acuity, days from AK onset (baseline) to raised pressure, and baseline and final BCVAs. Associated clinical findings are also included for these treatment groups including proportions with topical steroid use; those with closed anterior chamber angles; those with no cataract or pupil abnormalities; and those with mature cataract alone, dilated pupil alone, and dilated pupil with mature cataract.

Table 3 summarizes the outcomes, assessed by control of glaucoma, loss of the eye, and complications, in surgically treated glaucoma cases with and without a GDD. In the subset of 4 patients treated without a GDD, the outcomes were poor, leading to phthisis, evisceration, or failure to control pressure in the 3 cases for which outcome data were available. In those treated with a GDD, failure to control IOP was uncommon, occurring in 2 of 20 (10%), although additional AGMs were often required to achieve this (median of 2 for all GDDs combined shown in the last row of Supplemental Digital Content 4; see Supplemental Table 2, http://links.lww.com/ICO/B842). In GDD-treated patients, tube exposure occurred in 5 of 20 (25%) and revision surgery (not including tube exposure) in 4 of 20 (20%). Analysis of success rates for pressure control comparing glaucoma surgery with and without a GDD suggests a higher success rate with the use of a GDD (*P* = 0.006), with a point estimate of 90% for the difference in outcomes for those treated with a GDD (95% confidence limits 77%–100%). There were insufficient data to perform multivariable analyses of other factors that might have affected outcomes.

**TABLE 3.** Glaucoma Surgery Outcomes and Complications in 24 Eyes Both Without and With GDDs

Surgical Techniques		Outcomes and Complications					
		n	Failure*	Phthisis	Evisceration	Postsurgical Complications†		
**All surgeries without GDD combined**	4	1 (25%)	1 (25%)	1 (25%)	0		
Cyclodiode laser alone	2	0	1 (50%)	1 (50%)	0		
Trabeculectomy alone‡	1	No data	No data	No data	No data		
Trabeculectomy and cyclodiode laser	1	1 (100%)	0	0	0		
					**Tube exposure**	**Revision surgery**	**Complications combined**
**All GDD (tube) surgeries combined**	20	2 (10%)	0	0	5 (25%)	4 (20%)	9 (45%)
Drainage device alone	5	0	0	0	1 (20%)	0	1 (20%)
Drainage device with removal of ripcord	6	1 (17%)	0	0	2 (33%)	1 (17%)‖	3 (50%)
Drainage device with removal of ripcord and cyclodiode	7	0	0	0	2 (29%)	2 (29%)§	4 (57%)
Drainage device and cyclodiode	2	1 (50%)	0	0	0	1 (50%)||	1 (50%)

Analysis of the Difference in Outcomes Between Surgical Treatment Groups					
Treatment Group	n	Failure* (Including Phthisis and Evisceration)	Success n (%)	2-Sided Fisher Exact Test	Point Estimate for the Difference
All surgeries without GDD	4	3 (1 patient missing outcome data)	0	*P* = 0.006	90% (95% confidence limits 77%–100%)
All GDD surgeries combined	20	2¶	18 (90%)		

Percentages are of valid totals excluding missing values.

* Failure was defined as IOP >21 mm Hg despite additional AGM, or not reduced by 20%.

† There were no postsurgical complications for tube devices other than tube exposure or revisions, and no patients undergoing revisions happened to have tube exposure.

‡ Only 1 had a trabeculectomy alone, and it was performed abroad from where no outcome data were available.

§ One required adjustment of the ripcord before its removal, and the other required revision of the tube before keratoprosthesis.

‖ Trimming of tube length.

¶ Two patients failed the IOP criterion with an IOP of 25 mm Hg at the end of treatment.

## DISCUSSION

This study suffers from being retrospective in that it is possible that some cases of both glaucoma or OHT and AK-affected eyes may have been omitted because of data collection failures. In addition, the number of patients treated with surgery that did not include a GDD was small, because of these procedures being considered inappropriate for most patients, such that the comparison of outcomes we present has very limited value. In addition, the visual acuity outcomes have very limited value, as they are affected by both corneal opacity and keratoplasty. Although our simple analysis in Table 3 suggests that, for those requiring glaucoma surgery, there is a significant benefit to treating with a GDD as opposed to trabeculectomy, with or without cyclodiode laser, we accept that this finding is subject to numerous biases that we cannot control for. These include differences in the baseline data at the onset of AK, the point of development of glaucoma, and the degree of inflammatory activity at the time of glaucoma treatment, in addition to the complications associated with optical and/or TK.

Despite these limitations, we believe that it is reasonable to make some recommendations arising from the association of mature cataract and/or a fixed dilated pupil with the onset of AK-associated secondary glaucoma as well as a recommendation about the surgical approach when glaucoma has developed. Furthermore, our use of an existing database of AK incidence at our center permits probably the most reliable estimate of the incidence of secondary glaucoma in AK, an ultra-rare disease, while accepting that this incidence related to the past outcomes of AK management at our center and will vary by center depending on factors such as the efficacy of medical cures and TK rates; this is shown by the low rate of secondary glaucoma in a recently published Phase 3 trial of 127 patients, discussed further, in which there were no cases of secondary glaucoma.^11^

The outcome of AK-associated secondary glaucoma was poor in all but 1 study.^2^ In the case series described here, glaucoma was frequently associated with uncommon and severe AK complications (Table 1). These included scleritis requiring systemic immunosuppression^12^ in 4 of 40 (13%), keratoplasty in 34 of 40 (85%) of which 15 of 27 (56%) with known indications were therapeutic and (Table 2B) closed angles in 15 of 22 (68%) of assessable angles, with mature cataract and/or a fixed dilated pupil (also described as iris atrophy and paralytic mydriasis) in 26 of 40 (65%). Furthermore, over half the patients with glaucoma had developed this over 12 months from the onset of symptoms of AK (Table 1).

Mature cataract with or without a dilated pupil in association with AK was first described by Hertz et al.^13^ as “rapidly progressive cataract and iris atrophy during treatment of *Acanthamoeba* keratitis.” The incidence of a rapidly developing cataract that matured to a white cataract within 1–3 months was 9 of 81 (11%), of which 4 (5%) had a fixed dilated pupil (termed iris atrophy in the study).^13^ The focus of the discussion in the latter study was on the potential for antiamoebic treatment to have caused these complications; although glaucoma was found in 6 of 9 (66.6%), no association was observed with the presence of a fixed dilated pupil or mature cataract, possibly as glaucoma is common in severe AK cases treated with keratoplasty (8/9 of the cases in their report). The incidence of mature cataract and a fixed dilated pupil in AK reported in that series is probably substantially higher than that seen in recent years. In the recently published prospective randomized controlled trial of a new treatment for AK that included 127 patients (from 2017 to 2021), of whom 110 were cured without surgery within 12 months, only 2 (1.6%) developed raised IOP and only 1 of 112 (0.9%) developed cataract; no pupil-related abnormalities were reported.^11^ Another study describing a fixed dilated pupil (termed paralytic mydriasis) in AK was in a series describing the outcomes of TK, in which 18 of 32 (56%) had this finding and 9 of 32 (28%) underwent TK combined with cataract surgery.^9^ In our series of 40 eyes, TK was common in 16 of 28 (57%) confirming that association.

The association of a fixed dilated pupil and/or mature cataract with the onset of glaucoma is a striking finding in this study suggesting that these clinical associations herald the onset of glaucoma and that they might underlie the pathogenesis. This is the first study to report this association, which has implications for both pathogenesis and prophylaxis of glaucoma in AK when associated with mature cataract. A mature cataract, resulting in swelling of the lens to the point of angle closure, is a major cause of secondary angle closure glaucoma (phacomorphic glaucoma).^14,15^ Furthermore, the fixed dilated pupil in Urrets-Zavalia syndrome may also cause angle crowding and secondary angle closure glaucoma,^16^ and this is despite the fact that, in the authors' experience and as shown by the images in these reports,^13,16^ the degree of dilation is often less extreme than that in a AK-associated fixed dilated pupil. Keratitis-associated uveitis might also have contributed to secondary angle closure with anterior synechiae; although this occurs in uveitic glaucoma,^17^ it has rarely been reported as a complication of microbial keratitis with the exception of 1 previously reported case with AK^3^ and in some with syphilitic interstitial keratitis.^18,19^ In a series of 52 cases of microbial keratitis-associated glaucoma, it was acknowledged that, because of difficulty viewing, angle closure could not be excluded as a cause in some cases.^20^ The evidence for uveitis as an additional cause of angle closure in our series, as opposed to the effects of a mature cataract and fixed dilated pupil on the angle, is limited; this may be because the mechanisms of angle closure in uveitic glaucoma are different.^21^ Given these potential causes of angle closure in AK, it is reasonable to hypothesize that both are implicated in the causation of a high proportion (above 50% in our series). It is possible that ignoring the definitive treatment of mature cataract alone in 8 of 40 eyes (20%) or of mature cataract with a fixed dilated pupil in 13 of 40 (33%) by lens extraction, largely because of the difficulty performing cataract surgery in the presence of AK, has led to the preponderance of this uncommon clinical finding being associated with secondary glaucoma both in our series and in the other studies described above.^9,13^ The development of a mature cataract and/or a fixed dilated pupil should prompt a glaucoma assessment and treatment plan for the patient including anterior segment ocular coherence tomography or ultrasound biomicroscopy to assess the angle anatomy,^22,23^ before the onset of raised IOP. Furthermore, not all cases with AK, although still on treatment, are actively inflamed at the time when a cataract matures, which takes 1 to 3 months; in such cases, cataract surgery should be considered, despite the difficulties of surgery either through a variably opaque cornea or combined with a penetrating keratoplasty. Cataract surgery at this stage can be expected to reduce angle crowding and formation of peripheral anterior synechiae and has been shown in non-AK glaucoma case series to result in greater visual improvement and IOP control.^24^ Because of the poor outcomes of TK in AK, keratoplasty is best avoided where possible, but the outcomes of secondary glaucoma are also very poor and a combined cataract extraction and TK is probably a better option than delaying this until angle closure glaucoma is established.

How to successfully manage AK-associated secondary glaucoma once it has developed is unclear from our series alone because of the biases inherent in this study. Although the visual outcomes for those treated with AGMs alone were worse than those treated with glaucoma surgery, this finding may well have been due to these having more severe AK contraindicating surgery. We have shown that cyclodiode laser surgery alone is probably inadvisable. Eye inflammation at the time of glaucoma surgery, leading to a high risk of trabeculectomy failure, is why so few cases in our series received trabeculectomy. Those treated with GDDs had the best outcomes from the perspective of controlling IOP in 18 of 20 eyes and good visual outcomes in 7 of 20 (35%), but with a requirement for additional glaucoma surgery in 9 of 20 (45%). Other studies have made use of GDDs or recommended their use in this situation. The publication reporting outcomes for 17 cases of AK secondary glaucoma, with similar outcomes to 35 AK cases without glaucoma, used glaucoma surgery in 13 of 17 cases (3 with trabeculectomy, 9 with GDDs, and 1 with cyclophotocoagulation).^2^ The 1 publication (a study of 6 eyes) with a discussion focused on methods for managing these cases provides an analogy with uveitis-associated secondary glaucoma in which angle closure is common and GDDs are probably the optimal method of IOP control.^3^ Taking these 3 studies together, we concur with the advice given in the latter study that “medical therapy can be a temporary solution, but that in advanced AK, GDDs are needed.” However, despite access to glaucoma surgeons with a substantial experience of tube surgery, GDD success in our series frequently required additional surgery.

Prevention is better than cure. Probably the most major impact on glaucoma prophylaxis in AK patients is the adoption of newly described treatment, than can provide high cure rates without a TK in less than the 12 months taken for many AK glaucoma cases to develop.^11^ A further reduction in glaucoma cases can be expected by promptly reducing the risk of angle closure at the onset of a rapidly maturing cataract through cataract surgery performed before the onset of angle closure. If secondary glaucoma has developed and the angle is thought to be closed, then a GDD is probably the optimal first-line treatment.

## Supplementary Material
