# Supplementary material for: Acanthamoeba Keratitis Secondary Glaucoma Associated With Mature Cataract and a Fixed Dilated Pupil in a 40-Eye Series
Source: Cornea. 2025 Jun 19;45(6):748–53. doi: 10.1097/ICO.0000000000003918 (PMC13137970; doi:10.1097/ICO.0000000000003918)
Supplement: Supplementary file 1 [file cornea-45-748-s001.pdf]

Appendix A summary of key findings from studies describing glaucoma associated wth Acanthamoeba keratitis (AK); single case reports have not been included

Studies describing glaucoma associated with Acanthamoeba keratitis (AK): single case reports have not been listed

Study and year in reverse chronological order	Methodology and dates	Total number of eyes & subjects in study	Aims	Inclusion criteria	Therapeutic (T), Optical (O) Penetrating (P), Lamellar (L) Keratoplasty (K): TPK, OPK, TLK & OLK). Unspecified type: TK or OK	Numbers with Ocular hypertension	Numbers with Glaucoma	Status at onset of OHT or glaucoma (0= No, 1=Yes)										Associations	Proposed mechanism	Time from AK diagnosis to IOP rise (days)	Mean max. IOP	Number on Anti-glaucoma medication (AGM) alone	Number having glaucoma surgery with or without a glaucoma drainage device (GDD)	Complications	Final Best corrected visual acuity (BCVA)	Final Intra-ocular pressure (IOP)	Comment
								Topical steroid use	AK without previous eye disease and without keratoplasty	AK with previous glaucoma	After Keratoplasty not defined as therapeutic or optical	After TKP	After OKP	Keratoplasty done timing uncertan in relation to glaucoma	Asociated or with mature cataract	Associated with fixed dilated pupil (also known as iris atrophy and paralytic mydriasis)	Onset after/with cataract surgery										
**Large case series (≥20) of AK focused on describing AK in glaucoma**																											
Hossein R et al 2024 (current study)	Retrospective case series of glaucoma cases having Acanthamoeba keratitis from all Acanthamoeba cases 2000-2020	40 eyes in 39 cases	To descibe clinical characteristics and treatment outcomes	All glaucoma cases with AK	34 total in 40 eyes: 15 TK, 12 OK and 7 unknown indication	None	40 eyes	38/40 eyes after AK diagnosis	6/40 eyes	None	7	15	12	None	21	18	Not reported	Mature cataract and/or fixed dilated pupil (also known as iris atrophy and paralytic mydriasis	Secondary angle closure	Median 403 days (range 19-8984)	Not reported	16/40 eyes	24/40 eyes	**Surgery without a GDD** (n=4): 1 IOP control failures, 1 phthisis and 1 evisceration, 1 no data **GDD surgery (n=20):** 2 IOP control failures, 9 GDD complications requiring revision	**All groups n = 40 combined (glaucoma meds alone, glaucoma surgery, all with or without a keratoplasty)**: 8/39 ≥ 6/60, 31/39 <6/60, 1 missing data	**All groups combined**: median 13 mm Hg (range 0-50)	Final BCVA was worst in thoe treated with AGM alone. For thiose treated with surgery the group having a GDD had statistically significantly better crude outcomes than those treated with othher forms of surgery although this finding is subject to potentialo biases.
Al Owaifeer AM, 2021	Retrospective consecutive AK case series from King Khaled Hospital, Saudi Arabia from 2000-2018.	52 eyes in 52 patients	To report incidence of Ocular Hypertension (OHT) and glaucoma in AK and assocaite risk factors for these. Note some with OHT may have had glaucoma but could no be classified because disc damage and fields could not be assessed	Clinical features of AK with IVCM, histology of culture.	26 had PK those with TPK not stated	10/52 (19.2%)	17/52 (32.7%) overall but of whom 10 had had a PK.	3/52 (63.5%	Not reported	Not reported	10 of 17(59%) with glaucoma had a PKP	Not reported	Not reported	Uncertain	Not reported	Not reported	8/52 (15.4%)	Multivariable analysis including Sex , presenting BCVA, Disease stage, Steroid use, Adjuvant therapy and Having a PKP showed steroid use as the only statistically significant risk factor for the development of glaucoma p-value 0.049	Not discussed for glaucoma	8.4±16.6 months (range 1-76 months)	Not reported	4/17 (23.5%)	13/17 (76.5%) of whom 3 trabeculectomy, 9 glaucoma drainage device (GDD), 1 cyclocryoablation	Not reported	No difference between those with glaucoma and without in final BCVA with an overall improvement from baseline	ND	17 case glaucoma in AK series and the only one showing good outcomes of whom over half (9/17) had GDD
Kelley, 2006 Note: probably the same data as in Dossey A 2004	Retrospective consecutive case series 1994-2003	20 eyes in 20 patients	To describe glaucoma associated with AK	All AK cases in this period (confocal or culture+) with no previous history of glaucoma who developed glaucoma secondary to AK diagnosis and treatment and having 6 months follow-up. Subjects developing glaucoma after a TPK were excluded.	None (TPK was an exclusion criterion)	Not reported	6 of 20 (30%)	Not reported	Yes	Excluded	Excluded	Excluded	Excluded	Excluded	Not reported	Not reported	Not reported	Not reported	Pathology suggests angle closure as the probable mechanism	MEAN 161 (Range 60-540)	34 (Range 26-45)	2/6	3/6 had GDD (1/3 Ahmed replaced by Baerveldt; 1/3 Ahmed; 1/3 Molteno)	Hypotony with the Molteno tube required revision	1 20/70 after Ahmed then Barerveldt GDD ; 1 20/40 after Ahmed valve and PK; 3 enucleated; 1 LP with pthysis		This incidence is very high (30% of AK with glaucoma) and may reflect the referral pathways to this tertiary centre.GDD may be best option and non pupil block angle closure may be a cause
**Case series describing the abrupt onset of cataract with or without a fixed dilated pupil**																											
Herz, 2008	Retrospective case series of microbiologically confirmed AK cases 1995-2004 having available clinical records	81	Primary outcome was cataract in 9/81 laboratory confirmed AK cases. 6/9 developed rapid onset cataract with iris atrophy. Cataract developed within 4-15 weeks of AK diagnosis. Diagnostic delay not given. Iris atrophy ocurred within 6 months.	Cataract development in AK	8/9 had keratoplasty. Timing ok keratoplasty in relation to iris atrophy uncertain.	Not reported	6/9 developed secondary glaucoma of whom 4 had iris atrophy and 2 a cyclitic membrane.	1	Not reported	Not reported	Not repoorted	Not reported	Not reported	8/9	Not reported	4/81 (5%) overall and 4/9 (44%) of those with glaucoma	Not reported	Iris atrophy 4/6 and cyclitic membrane in 2/6. Status of anterior chamber angle uncertain	No comment	Not reported	Not reported	Not reported	3 trabeculectomy and 1/3 later had a GDD	1 enucleated for pain	1 CF, 1 Enucleated, 2 at 20/40, 1 at 20/125 and 1 at 20/25	Not reported	Abrupt onset of cataract described. Cataract developed within 1-3 months. In 3/9 cases cataract developed within 2 months of onset of severe inflammation. Timing of keratoplasties uncertain and possibility of Urrets-Zavailia syndrom not discussed. **Good photographs**
**Case series of therapeutic keratoplasty for AK with glaucoma described in which data has been provided on glaucoma**																											
Roozbahani M, 2019	Retrospective review of all AK subjects diagnosed at Wills Eye Hospital, Philadelphia from January 2009 to February 2016	63	To identify whether risk factors at presentation could predict the need for TKP amongst a series of 63 AK cases	TPK	12	Not reported	3/12 (25%)	Not reported	Not reported	Not reported	Not repoorted	2	Not reported	Not reported	ND	Not reported	Not reported	ND	No comment	Not reported	Not reported	Not reported	Not reported	Not reported	Not reported	Not reported	2 had tube surgery, 1 cyclocryotherapy
Kitzmann, 2009	Retrospective cases series of AK cases having a keratoplasty at a single centre between 1/1/1980 to 31/12/2007	31 eyes in 30 subjetcs	To report outcomes of TPK and OPK at a single centre	All cases with histological confirmation of AK treated having a keratoplasty	20 TPK, 2 TLK, 8 OPK, 1 OLK	Excluded	8/31 (26%)	Not reported	Excluded	Excluded	8	7/22 (32%)	1/9 (11%)	None (all after keratoplasty)	Not reported	Not reported	2 cataract surgery at time of OKP and 4/22 (18%) in TPK group and 1/11 on OPK group after keratoplasty. Cataract maturity not described.	All eyes apart from 3 having OKP were on anti-amoebic treatment at tme of keratoplasty. Repeat keratoplasty in 12/22 (55%) TPK and 1/9 (11%) OPK.	No comment	Not reported	Not reported	7/22 (32%) TPK and 1/9 (11%) OPK	1/22 (5%) TPK	Not reported	Not reported for glaucoma subset	Not reported	7/22 (32%) TPK for AK developed glaucoma one of whom had glaucoma surgery versus 1/9 (11%) OPK none needing glaucoma surgery.
Kashiwabushi, 2008	32 patients with AK having a TPK between 08/1996 and 08/2005	32 subjects 32 eyes	To report corneal transplant survival and visual outcomes after TPK for AK	TPK subjects in the study sime period. AK confirmed by smears or cultures	32 eyes with TPK	Not reported	13/32 (40%)	Not reported	Excluded	Not reported	Not repoorted	All subjects (eyes) 13/32 (40%)	None	All developed glaucoma after TPK	Not reported	18/32 (56%) reported with paralytic mydriasis	9/32 (28%) had combined cataract surgery. Cataract maturity not described.	Glaucoma was common following TPK with 13/32 (40%) developing glaucoma after TPK.TPK with glaucoma transplants failed significantly more often.	Not discussed for glaucoma	Not reported	Not reported	Not reported	Not reported	Not reported	Not reported	Not reported	Corneal transplants failed significantly more often after the development of glaucoma. Paralytic mydriasis (fixed dilated pupil) was common in 18/32 (56%) but not associated with graft failure.
**Comparison of optical and therapeutic keratoplasty outcomes for AK**																											
Awwad, 2005	Retrospective case series 01/1995 to 09/2004	13 eyes in 13 subjects	To assess results of an optical PK for AK.	AK (diagnosed by culture and/ or IVCM and or biopsy) having an OPK. All eyes had been followed up for at least 3 months after discontinuation of anti-amoebic therapy before OPK.	13 Optical keratoplasties after discontinuation of anti-amoebic treatment	Not reported	5/13 had glaucoma before OPK surgery	All patients	Excluded	5	None	Excluded	None	All OPK done after glaucoma diagnosed. None developed glaucoma who did not have it before OPK	None reported	None reported	None had mature catract reported but 4/5 (80%) glaucoma cases had cataract extraction with OPK	Secondary glaucoma before OPK. No cases developed secondary glaucoma after OPK unless glaucoma was present at the time of OPK	Not reported	Not reported	21.4(range 16-29) whilst on 1-3 glaucoma drugs	4/5 after OPK	1/5 had OPK complicated by loss of control of glaucoma requiring a GDD and regraft; 1 had trabeculectomy after OPK; 1 had OPK combined with a GDD, and 1 a valve revision with OKP. 1 had cyclodiode laser before OPK	5/13 cases with secondary glaucoma all deteriorated after OPK; the 8/13 without secondary glsucome did not develop glaucoma	12/13 ≥20/40; 1/5 with glaucoma had LP only	ND but 4/5 had adjunctive glaucoma medications 6 months after the OPK	Presence of secondary glaucoma before an OPK after AK requires careful evaluation and may be better with combined OPK and glaucoma surgery

Large (more than 50 subjects or eyes) AK case series with data on glaucoma

	**Brief summary**
Duguid, 1997	Consecutive AK case series. 23/111 had a poor outcome of whom 4 had glaucoma 2 of whom were enucleated. It's unclear if others may have had secondary glaucoma.
Ficker, 1993	AK transplant case series of 13 penetrating keratoplasties in 11 subjects. Glaucoma was seen within 3 months of graft surgery in all four patients (4 of 13 PK's in 11 patients), only one of whom had had perforation before PK. Of the four patients in whom glaucoma developed, one refused surgery and three underwent trabeculectomy, two of whom had additional tube drainage procedures. One graft failed due to uncontrolled glaucoma."
Bacon, 1993	AK consecutive case series 1984-1992. Glaucoma numbers calculated from percentages 10/77 (13%) eyes (72 patients). Of 15/73 with poor outcomes VA <6/18, 5/15 had glaucoma
