# Supplementary material for: Acanthamoeba Keratitis Secondary Glaucoma Associated With Mature Cataract and a Fixed Dilated Pupil in a 40-Eye Series
Source: Cornea. 2025 Jun 19;45(6):748–53. doi: 10.1097/ICO.0000000000003918 (PMC13137970; doi:10.1097/ICO.0000000000003918)
Supplement: Supplementary file 2 [file cornea-45-748-s002.pdf]

Supplemental Table 1

Annual incidence of glaucoma or ocular hypertension (OHT) in *Acanthamoeba* keratitis affected patients and eyes from 2000-2015

Year	Total number of eyes with AK	Total number of patients with AK	Total number of AK eyes with OHT or glaucoma (percent)
2000	8	8	1 (12.5%)
2001	8	7	0
2002	7	7	0
2003	10	10	1 (10%)
2004	21	20	0
2005	17	16	1 (5.9%)
2006	18	17	1 (5.6%)
2007	19	16	1 (5.3%)
2008	15	15	0
2009	17	16	0
2010	24	23	5 (20.8%)
2011	39	36	3 (7.7%)
2012	42	41	2 (4.8%)
2013	65	65	6 (9.2%)
2014	56	54	1 (1.8%)
2015	51	47	4 (7.8%)
**Total**	**417**	**398**	**26 (6.2%)**
