# Supplementary material for: Acanthamoeba Keratitis Secondary Glaucoma Associated With Mature Cataract and a Fixed Dilated Pupil in a 40-Eye Series
Source: Cornea. 2025 Jun 19;45(6):748–53. doi: 10.1097/ICO.0000000000003918 (PMC13137970; doi:10.1097/ICO.0000000000003918)
Supplement: Supplementary file 3 [file cornea-45-748-s003.pdf]

Supplemental Table 2

Outcomes of glaucoma treatments for 40 eyes with Acanthamoeba keratitis between 1992-2020 with associated clinical findings in the right had section of the Table. Subsections, headed in bold typeface, are for all treatments combined and for mutually exclusive subsets of these treatments. Percentages are of valid totals excluding missing values.

		**OUTCOMES FOR GLAUCOMA TREATMENTS and the numbers of anti-glaucoma medications (AGM)[a] used**								**ASSOCIATED CLINICAL FINDINGS n (%)**					
	**N**	**Visual acuity (Snellen metres)**					**IOP: median (range)**		**AGM[a]**						
		Baseline[b] BCVA[c] n (%)		Final BCVA n (%)											
**GLAUCOMA TREATMENT GROUP with and without keratoplasty**	**40**	Better than or equal to 6/60	Worse than 6/60	Better than or equal to 6/60	Worse than 6/60	Days from symptom onset to raised IOP: median (range)	IOP[d] at baseline[b] in mmHg	Final IOP in mmHg	Number of agents: median (range)	Topical steroid use after AK diagnosis	Closed angle[e]	No cataract or pupil change	Mature cataract alone	Dilated pupil alone	Dilated pupil with mature cataract
**ALL treatments combined**	**40**	16 (53%)	14 (47%)	8 (21%)[f]	31 (79%)	403 (19-8984)	15 (6-24)	13 (0-50)[ghi]	2 (0-5)[hi]	38 (95%)	15 (68%)	14 (35%)	8 (20%)	5 (13%)	13 (33%)
Missing values n		*10*		*1*		*12*	*14*	*1*	*3*	*0*	*18*	*0*	*0*	*0*	*0*
**AGM alone: no keratoplasty**	**5**	2 (50%)	2 (50%)	0	5 (100%)	105 (49-166)	11 (10-18)	18 (12-42)[i]	3 (0-5)[i]	4 (80%)	1 (100%)	1 (20%)	1 (20%)	1 (20%)	2 (40%)
Missing values n		*1*		*0*		*1*	*4*	*1*	*1*	*0*	*4*	*0*	*0*	*0*	*0*
**AGM alone: with keratoplasty**	**11**	2 (25%)	6 (75%)	1 (10%)	9 (90%)	581 (86-8984)	15 (6-24)	20 (5-50)[h]	1.5 (0-4)[h]	11 (100%)	4 (67%)	4 (36%)	3 (27%)	1 (9%)	3 (27%)
Missing values n		*3*		*1*		*3*	*1*	*0*	*1*	*0*	*5*		*0*	*0*	*0*
**Glaucoma surgery: no keratoplasty**	**1**	1 (100%)	0	0	1 (100%)	ND[j]	ND	25	2	1 (100%)	ND	0	0	0	1 (100%)
Missing values n		*0*		*0*		*1*	*1*	*0*	*0*	*0*	*1*	*0*	*0*	*0*	*0*
**Glaucoma surgery: with keratoplasty**	**23**	10 (59%)	7 (41%)	7 (30%)	16 (70%)	434 (19-5956)	16 (8-23)	12 (0-25)[g]	1.5 (0-4)	22 (96%)	10 (67%)	9 (39%)	4 (17%)	3 (13%)	7 (30%)
Missing values n		*6*		*0*		*7*	*8*	*0*	*1*	*0*	*8*	*0*	*0*	*0*	*0*
Cyclodiode laser alone	**2**	0	2 (100%)	0	2 (100%)	471 (471)	12	1 (0-2)[g]	0 (0)	1 (50%)	2 (100%)	0	1 (50%)	0	1 (50%)
Missing values n		*0*		*0*		*1*	*1*	*0*	*0*	*0*	*0*	*0*	*0*	*0*	*0*
Traby[k] ± cyclodiode	**2**	1	0	0	2	ND	16	30.5	3	2 (100%)	1 (100%)	0	1 (50%)	0	1 (50%)
Missing values n		*1*		*0*		*2*	*1*	*0*	*0*	*0*	*1*	*0*	*0*	*0*	*0*
All GDD[l] combined[m]	**20**	10 (67%)	5 (33%)	7 (35%)[n]	13 (65%)	397 (19-5956)	16 (8-23)	12 (1-25)[o]	2 (0-4)[p]	20 (100%)	7 (58%)	9 (45%)	2 (10%)	3 (15%)	6 (30%)
Missing values n		*5*		*0*		*5*	*7*	*0*	*0*	*0*	*8*		*0*	*0*	*0*

[a]AGM: anti-glaucoma medication; [b] Baseline: at time of AK diagnosis; [c] BCVA: Best-Corrected Visual Acuity; [d] IOP: Intraocular pressure; [e] Closed angle: more than 180° of iridotrabecular contact; [f] Individual visual acuities: 6/7.5, 6/9, 6/12, 6/15, 6/18, 6/18, 6/24, 6/36; [g]one eye was phthisical [IOP zero at that point] and another eviscerated [IOP 2mm at that point]; [h]one eye was enucleated due to panophthalmitis (values are immediately prior to this); [i]Two eyes were eviscerated (values are immediately prior to this; [j]ND: No/missing data; [k]Traby:trabeculectomy of which 1 patient had additional cyclodiode resulting in uncontrolled IOP leading to no light perception; [l]GDD: Glaucoma Drainage Device; [m]All GDD include GDD alone (n=5), GDD with ripcord out (n=6), GDD with ripcord out and cyclodiode (n=7), GDD with cyclodiode (n=2); [n]Individual visual acuities: 6/9, 6/12, 6/15, 6/18, 6/18, 6/24, 6/36; [o]Two patients had IOP above 21 mmHg at the end of treatment; one of our criteria for failure; [p]Six patients (30%) were on no AGM
