# Supplementary material for: Acanthamoeba Keratitis Secondary Glaucoma Associated With Mature Cataract and a Fixed Dilated Pupil in a 40-Eye Series
Source: Cornea. 2025 Jun 19;45(6):748–53. doi: 10.1097/ICO.0000000000003918 (PMC13137970; doi:10.1097/ICO.0000000000003918)

Supplemental Figure 1 Acanthamoeba keratitis patients numbers seen each year or period at Moorfields Eye Hospital from 1984 to 2019.

794 patients were recorded in the period 1992-2019; a period covered by the Acanthamoeba keratitis secondary glaucoma study.

Data collection methodology varied for different periods (a-e) and are not directly comparable. These were as follows:

a) 1984-1996 data was published in a letter as a bar chart[1] and was collected using the clinical and/or microbiological (culture or histology) criteria used in the previous study,[2]

b) data for the period 01-10-1997 to 30-09-1999 was available from a national survey[3] for which annual figures are not available: there were 37 cases in this period.

c) from 2000 to April 2012 cases were identified from our current microbiology laboratory electronic database, and an electronic letter search of our electronic patient database, both of which started in 2000. Criteria for inclusion were a positive *Acanthamoeba* culture, histopathological confirmation of trophozoites and/or cysts, culture-negative cases shown to have *Acanthamoeba* cysts on confocal microscopy, and those with a typical clinical course and response to treatment.

d) from March 2012 to December 2013 cases were identified prospectively as part of studies being carried out on AK using the (c) criteria

e) from 01-01-2014 to 31-12-2019 cases were identified by retrospective audit, using the same criteria as for (c) but with the addition of *Acanthamoeba* DNA identification by polymerase chain reaction (PCR) as an additional inclusion criterion (black). The data from 1984-2016 has been published.[4]

1. Morlet N, Duguid G, Radford C, et al. Incidence of acanthamoeba keratitis associated with contact lens wear. Lancet 1997;350:414.
2. Radford CF , Bacon AS, Dart JK, et al. Risk factors for acanthamoeba keratitis in contact lens users: a case-control study. BMJ 1995;310:1567–70.
3. Radford CF , Minassian DC, Dart JK. Acanthamoeba keratitis in England and Wales:incidence, outcome, and risk factors. Br J Ophthalmol 2002;86:536–42
4. Carnt N J et al. *Acanthamoeba* keratitis: confirmation of the UK outbreak and a prospective case-control study identifying contributing risk factors. Br J Ophthalmol 2018; 102(12): 1621-1628.

Year	Patients
1984-88	17
1989	3
1990	11
1991	22
1992	20
1993	42
1994	46
1995	38
1996	16
1997-99	37
2000	8
2001	7
2002	7
2003	10
2004	20
2005	16
2006	17
2007	16
2008	15
2009	16
2010	23
2011	36
2012	41
2013	65
2014	54
2015	47
2016	59
2017	63
2018	43
2019	32
Total	847

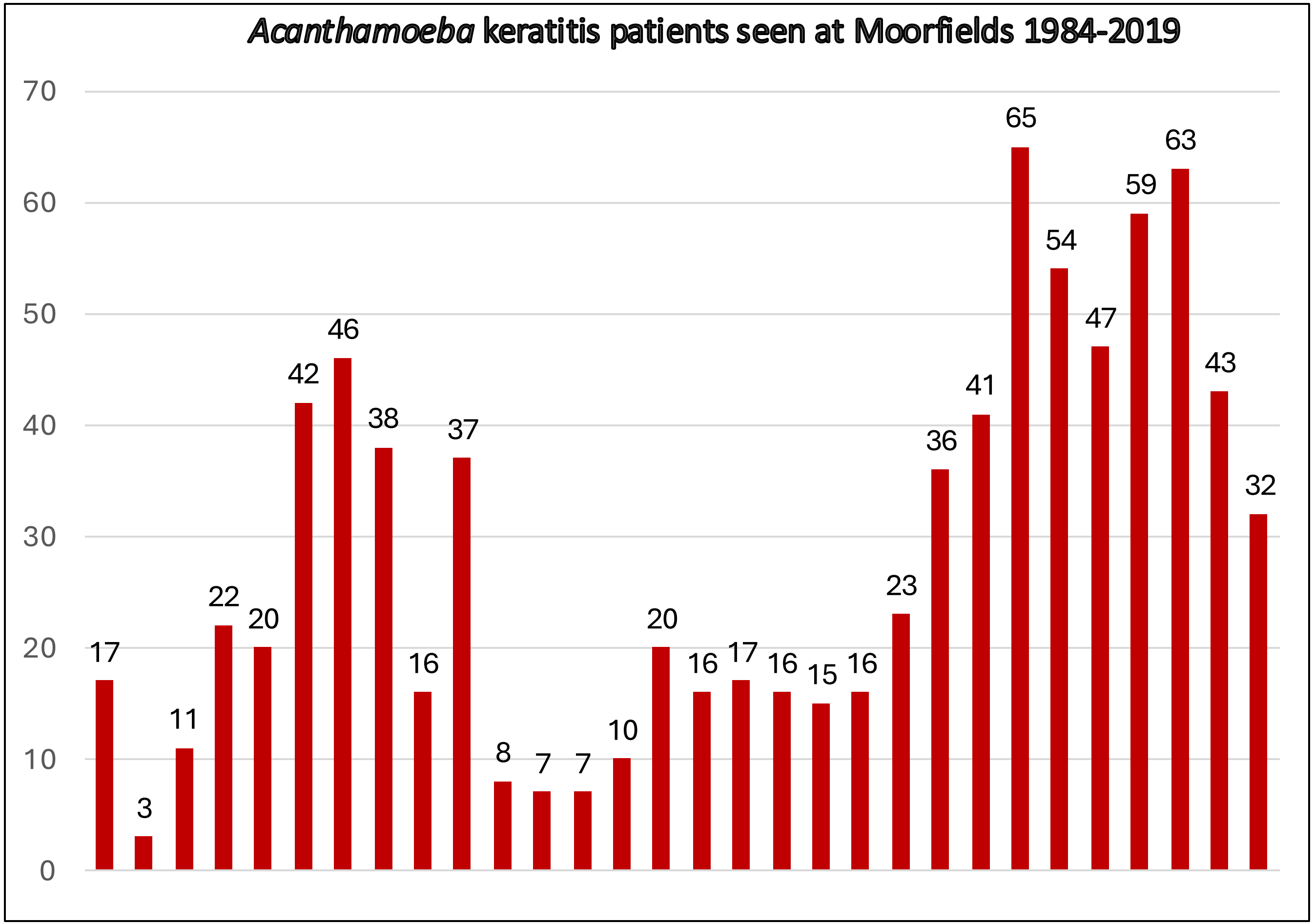

Supplement: Supplementary file 4 [file cornea-45-748-s004.pdf]
